# Computed Tomography Assessment of Diaphragm Thickness in Myasthenia Gravis With Clinical and Functional Correlations

**DOI:** 10.7759/cureus.110567

**Published:** 2026-06-09

**Authors:** Evgeny B Topolnitskiy, Valerii V Gusakov, Alex A Volinsky

**Affiliations:** 1 Thoracic Surgery, Siberian State Medical University, Tomsk, RUS; 2 Mechanical and Aerospace Engineering, University of South Florida, Tampa, USA

**Keywords:** clinical risk stratification, ct (computed tomography) imaging, diagnostic imaging protocols, diaphragm thickness, myasthenia gravis (mg), neuromuscular disorders, respiratory muscle involvement, severe respiratory failure, structural-functional correlations, thoracic ultrasonography

## Abstract

Myasthenia gravis (MG) is an autoimmune disorder of the neuromuscular junction in which respiratory muscle involvement, particularly of the diaphragm, constitutes the principal risk factor for myasthenic crisis and respiratory failure. Objective structural evaluation of the diaphragm in MG remains incompletely standardized. This narrative review synthesizes published evidence on diaphragm thickness measurement in generalized MG, with attention to measurement reproducibility, normative reference values, structural-functional linkages, and potential clinical applications of computed tomography (CT). Studies were identified through a targeted search of peer-reviewed databases covering neuromuscular imaging, diaphragm physiology, and respiratory failure, with emphasis on MG. Studies reporting diaphragm measurements with explicit methodological details or clinical outcomes were prioritized, in both MG cohorts and mixed populations, with particular attention to CT. A small number of studies have used diaphragm ultrasound in MG to assess increased fatigability and predict extubation failure. Direct CT-ultrasound matching in myasthenic patients is lacking; however, in non-myasthenic populations, CT measurements at the L1 vertebral level and at the celiac trunk are reproducible and show consistent associations with ultrasound-derived functional indices of diaphragm dysfunction. Left-sided CT measurements correlate more strongly with multiple functional parameters, whereas right-sided measurements show moderate associations with selected indices. Interobserver agreement for CT is good in non-myasthenic cohorts. Because the available structural-functional evidence derives almost entirely from non-myasthenic, mixed neuromuscular, ICU, or diaphragmatic paralysis cohorts rather than MG-specific populations, these findings are hypothesis-generating: CT-based diaphragm assessment is a candidate structural surrogate for functional impairment in MG and a potential adjunct to risk stratification and therapeutic monitoring. The evidence base remains limited by small cohorts and reliance on mixed disease populations. CT is therefore currently best regarded as complementary rather than definitive, and prospective MG-specific studies, stratified by the Myasthenia Gravis Foundation of America (MGFA) class and antibody subtype (acetylcholine receptor, muscle-specific kinase, low-density lipoprotein receptor-related protein 4, seronegative) and integrated with diaphragmatic compound muscle action potential, diaphragm ultrasound, forced vital capacity, and negative inspiratory force trends, are needed before firm clinical thresholds can be established.

## Introduction and background

The diaphragm is the primary respiratory muscle, responsible for generating 60-80% of tidal volume during spontaneous breathing [1,2]. In neuromuscular diseases, the degree of diaphragmatic involvement largely determines the severity of respiratory impairment, prognosis, and the need for intensive care intervention [3,4]. Among neuromuscular disorders, myasthenia gravis (MG) occupies a distinct position as the most prevalent autoimmune condition of the neuromuscular junction, characterized by fatigable muscle weakness that partially recovers with rest [5].

MG arises from an autoimmune attack directed against acetylcholine receptors (AChRs) or associated postsynaptic proteins at the neuromuscular junction [6]. The resulting impairment of neuromuscular transmission produces pathological fatigability, the clinical hallmark of the disease. Respiratory muscle involvement in generalized MG carries potentially life-threatening consequences; myasthenic crisis with respiratory failure occurs in 15-20% of patients within the first two years of disease onset [7].

The only available systematic review concluded that no definitive recommendation can be made regarding a preferential method of respiratory assessment of patients with neuromuscular diseases during weaning from mechanical ventilation [8]. Conventional methods for assessing diaphragm function in MG, including spirometry, transdiaphragmatic pressure measurement, and fluoroscopy, carry significant limitations: invasiveness, operator dependence, or inability to evaluate the structural integrity of the muscle directly [9]. Diaphragm ultrasound permits real-time assessment of thickness and contractile function but is characterized by considerable operator variability [10]. Computed tomography (CT), by contrast, provides highly reproducible quantitative evaluation of diaphragm structure with less operator dependence, representing a promising modality for standardized structural monitoring of respiratory muscles in MG [11].

Despite the clinical relevance of this question, a critical knowledge gap persists, since no standardized CT protocol for diaphragm assessment that is validated specifically in the MG population exists, and the relationship between CT-derived structural metrics and clinically meaningful functional outcomes in MG has not been characterized. The goal of this review is therefore twofold: to synthesize available evidence on CT-based measurement of diaphragm thickness and its structural-functional linkages in MG and to outline a proposed research framework for a candidate standardized measurement protocol that would require prospective MG-specific validation prior to clinical implementation. The authors draw on prior work documenting Quantitative Myasthenia Gravis Score (QMGS) dynamics as a predictor of myasthenic crisis following thymectomy, which provides relevant clinical context for the role of objective structural biomarkers in this population [12].

## Review

Literature search strategy

This narrative review was developed through a targeted search of peer-reviewed literature addressing CT evaluation of diaphragm thickness in neuromuscular diseases, with particular emphasis on MG. Searches were conducted across PubMed/MEDLINE, Scopus, and Cochrane databases using combinations of the following terms: myasthenia gravis, diaphragm, computed tomography, diaphragm thickness, respiratory muscle, neuromuscular disease, diaphragm ultrasound, and myasthenic crisis. No formal date restriction was applied; however, publications from 2010 onward were prioritized to reflect advances in CT imaging protocols, while earlier anatomical and physiological references were retained when methodologically essential. Inclusion criteria were: (i) original studies, case reports, or reviews reporting quantitative diaphragm thickness measurements by CT or ultrasound, or providing primary anatomical, physiological, or pathophysiological data on the diaphragm or neuromuscular junction with special concern on MG or related neuromuscular disease; (ii) explicit description of the measurement protocol or clinical outcome; and (iii) publication in English in a peer-reviewed source. Exclusion criteria were: the absence of explicit CT or ultrasound measurement protocol, duplicate populations, abstracts without full text, and studies unrelated to respiratory muscle structure or function. Studies were prioritized when they reported (a) MG-specific cohorts, (b) head-to-head CT-ultrasound comparisons, (c) reproducibility data, or (d) normative reference values. References to anatomy, physiology, and methodology were retained when foundational to the synthesis, even if older than 2010.

The database search identified 47 records. After title and abstract screening against the criteria above, 10 records were excluded (duplicate populations, n=4; absence of an explicit CT or ultrasound measurement protocol, n=4; topic unrelated to respiratory muscle structure or function, n=2), and 37 records proceeded to full-text assessment, all of which met the inclusion criteria and were incorporated into the synthesis.

As a narrative review, formal risk of bias scoring and a PRISMA flow diagram were not performed. This limitation is acknowledged in the manuscript. Data were synthesized descriptively, with correlation coefficients and reproducibility indices reported as extracted from individual source studies.

Neurologic context

MG is clinically heterogeneous, and any structural biomarker proposed for this population must ultimately be interpreted in relation to disease classification and serological profile. The Myasthenia Gravis Foundation of America (MGFA) clinical classification (I-V) stratifies patients by topographic distribution and severity of weakness, with classes IV and V capturing severe generalized and crisis-level respiratory involvement, respectively. Antibody subtype further conditions phenotype: AChR-positive MG (most common) is associated with a broad clinical spectrum and frequent thymic pathology; muscle-specific kinase (MuSK)-positive MG shows a predilection for bulbar and respiratory weakness and has some tomographic evidence of tongue atrophy; low-density lipoprotein receptor-related protein 4 (LRP4)-positive and seronegative subtypes constitute smaller, less well-characterized groups with variable respiratory phenotypes. Respiratory phenotypic heterogeneity therefore spans patients with predominantly generalized fatigable weakness, those with isolated or disproportionate diaphragmatic involvement, and those whose crisis risk is driven primarily by bulbar dysfunction and aspiration. In current clinical practice, respiratory monitoring in MG relies chiefly on forced vital capacity (FVC) and negative inspiratory force (NIF), serially tracked because of their established prognostic value: declining FVC trends and NIF less negative than approximately −20 to −30 cmH₂O are widely used thresholds prompting consideration of escalation or ventilatory support. Any future role for CT-derived diaphragm thickness in MG should therefore be evaluated as an adjunct to, rather than a replacement for, FVC and NIF trends and should be analyzed within MGFA- and antibody-stratified cohorts.

Measurement techniques

Based on published protocols, diaphragm thickness measurements were performed at two primary anatomical levels [13-15].

Celiac Trunk Level

On axial CT slices, hemidiaphragm thickness was measured at the level of the celiac trunk origin, at the intersection of the horizontal tangent to the anterior margin of the spinal canal with the lateral contours of each hemidiaphragm. Minimum thickness perpendicular to the diaphragm surface was recorded. Measurements on coronal reformats were also obtained [16-18].

L1 Vertebral Level

On coronal images, right and left diaphragmatic crural thickness was measured along the mid-body of L1. This level was selected to avoid superimposition of the psoas muscle encountered at more caudal vertebral levels [16-18]. Image acquisition parameters are listed in Table 1.

**Table 1:** Standard CT image acquisition parameters for diaphragm thickness measurement. This table represents an interpretive synthesis rather than a replication of any published figure or table. Adapted from studies by Ufuk et al. [18] and Lallement et al. [19].

Parameter	Specification
Scanner	64-slice or higher multidetector CT
Patient position	Supine, breath held at full inspiration
Tube voltage	100-120 kV
Tube current	90-300 mA with dose modulation
Pitch	0.98
Collimation	<0.625 mm
Slice thickness	1-1.5 mm
Reconstruction kernel	High-resolution (soft tissue)
Reformations	Axial and coronal at 1.5 mm intervals
Window settings	Mediastinal (center 90 HU; width 350 HU)
Contrast	Optional; both enhanced and non-enhanced acquisitions are acceptable

Pathophysiology in MG

MG represents the prototypical neuromuscular disorder [5]. Pathological fatigability becomes particularly dangerous when respiratory muscles are involved, since each successive effort produces a disproportionate decline in force generation and creates a real risk of abrupt respiratory decompensation [8]. Despite this clinical relevance, the diaphragm, the primary respiratory muscle, until recently remained largely unexplored in MG [20].

The mechanism of diaphragmatic involvement reflects a reduction in functionally available AChRs at the neuromuscular junction of diaphragmatic fibers [6]. This receptor loss contributes to atrophic changes in muscle fibers at both the cellular and molecular levels, a phenomenon demonstrated across several muscle tissues. In the diaphragm, electron microscopy in experimental autoimmune MG revealed simplification of the postsynaptic region due to degeneration of postsynaptic folds in the left hemidiaphragm, accompanied by a significant increase in the width of the primary synaptic cleft. These changes correlated with disease severity in rats [21]. In a genetic mouse model of congenital myasthenic syndrome (VAChT knockdown), investigators reported no gross morphological changes in the diaphragm neuromuscular junction but observed increased spontaneous synaptic vesicle exocytosis, reduced miniature endplate potential amplitude, and abnormally shaped synaptic vesicles that were flattened and elliptical, without impairment of resting respiratory mechanics [22].

On this basis, we hypothesize that morphological changes in the diaphragm can be detected by CT prior to clinical manifestation. This hypothesis builds on current knowledge from diaphragm ultrasound in MG, where clinically significant respiratory muscle fatigue is characterized by progressive dysfunction across repeated respiratory maneuvers, fundamentally distinguishing it from static weakness. This dynamic component complicates standard quantitative assessment and underscores the need for specialized diagnostic approaches [20,23].

Structural-functional correlations

Structural-functional correlations derive from non-myasthenic and mixed cohorts and are extrapolated to MG with appropriate caution. Lallement et al. demonstrated a significant relationship between ultrasound-confirmed diaphragmatic dysfunction and CT-measured diaphragm thickness in non-myasthenic disease [19]. Ma et al. compared 34 patients with generalized MG against 30 healthy volunteers, using ultrasound-derived functional parameters as the reference standard [23]. Several reports describe successful ultrasound measurement of diaphragm thickness in MG patients for weaning from mechanical ventilation [24-27]. Because diaphragmatic dysfunction is documented in that group, analogous structural-functional linkages on CT are biologically plausible. Results are reported separately for right and left hemidiaphragms because of marked anatomical asymmetry of the diaphragmatic crura [13-15]. The same asymmetry has been demonstrated in ultrasound for a myasthenic patient, but only as a case report [25].

Right-sided measurements

In non-myasthenic patients, right diaphragmatic crus thickness at L1 on CT correlated with deep-breathing excursion amplitude (r = 0.34; p < 0.01), voluntary sniff amplitude (r = 0.31; p < 0.05), and end-expiratory thickness (r = 0.26; p < 0.05). Right crus thickness at the celiac trunk level correlated with quiet-breathing excursion amplitude (r = 0.32; p < 0.05). These moderate associations indicate that CT and ultrasound capture partially distinct dimensions of diaphragm structure and function in patients without MG [19,28-30]. Most ultrasound measurements were performed on the right side [23,25-27]. In particular, the right diaphragmatic dome has also been studied using ultrasound in the study by Ma et al. in MG. They demonstrated significant dynamic changes in parameters during respiratory movements, consistent with respiratory muscle fatigue, compared to healthy volunteers [23]. No direct CT comparisons have been reported in MG, leaving CT assessment at the level of the crura as an open avenue for investigation. 

Left-sided measurements

Foundational studies of diaphragm atrophy in MG were performed on the left hemidiaphragm or on mixed cells from the entire diaphragm [21,22]. In non-myasthenic patients, left-sided CT measurements at L1 and at the celiac trunk showed moderate-to-strong correlations with all ultrasound functional parameters (p < 0.001), including quiet- and deep-breathing excursion amplitudes, voluntary sniff amplitude, end-expiratory and end-inspiratory thickness, maximal inspiratory thickness, and the diaphragm thickening fraction [19,28-30]. In the MG cohort of Ma et al., no data were reported for the left dome, reflecting the greater practical utility of left-dome ultrasound in routine clinical practice [23]. Only one identified report has clearly described the left hemidiaphragm in a myasthenic patient, which represents a limitation of the available ultrasound evidence [25].

Structural and functional differences between the right and left hemidiaphragms necessitate a laterally specific diagnostic approach. The right crus is thicker, stronger, and longer than the left, reflecting hepatic support and asymmetric functional loading [13-15]. If confirmed in MG-specific cohorts, dysfunction thresholds would likely need to be side-specific; none have yet been validated in MG [16,23]. In non-myasthenic cohorts, the right hemidiaphragm is more resistant to measurable structural change: CT reliably detects structural loss only with complete right-sided paralysis, whereas any degree of left-sided weakness is accompanied by measurable CT changes. Whether this pattern extrapolates to MG remains a hypothesis requiring prospective validation.

The stronger correlations observed between left-sided CT measurements and ultrasound functional parameters may partly reflect the more perpendicular orientation of the left hemidiaphragm relative to the ultrasound beam, improving the precision of the ultrasound reference standard on that side [28,29].

Dynamic fatigue assessment

A cardinal clinical feature of MG is progressive fatigability with repeated effort. Dynamic ultrasound assessment during serial deep breaths revealed progressive diaphragmatic fatigue in MG patients that was absent in healthy volunteers [23].

Although CT provides a static structural evaluation, the correlations described above in non-myasthenic cohorts raise the hypothesis that CT measurements may eventually help identify patients at heightened risk of respiratory failure in MG. Reduced baseline diaphragm thickness on CT, if it reflects atrophy from recurrent dysfunction, may serve as a candidate structural marker of more severe disease; this remains hypothesis-generating and is not yet supported by prospective MG-specific evidence [11,31]. Prospective studies are needed to define CT-based prognostic thresholds in MG.

Normative values and thresholds

Normative values and thresholds are derived from established evidence in non‑myasthenic cohorts. In healthy adults, CT measurements in Table 2 demonstrate a clear variation in the diaphragmatic pillar thickness according to the anatomical level and sex. 

**Table 2:** CT diagnostic thresholds for diaphragm dysfunction by side and measurement level, derived from non-myasthenic cohorts. These thresholds are not validated for MG and must not be applied clinically to MG patients without prospective MG-specific confirmation. Sensitivity and AUC values are expressed as rounded ranges summarizing diagnostic patterns described by Lallement et al. [19]. No right-sided thresholds for weakness have been validated; right-sided structural changes are measurable on CT only in the presence of complete paralysis. MG: Myasthenia gravis; AUC: area under the curve

Threshold, mm	Dysfunction type	Side	CT level	Sensitivity, %	AUC
<4.5	Paralysis	Right	L1	85-90	0.9-0.97
<3	Paralysis	Right	Celiac trunk	75-85	0.85-0.95
<3.8	Paralysis	Left	L1	85-95	0.95-1
<2.6	Paralysis	Left	Celiac trunk	90-100	0.95-1
<5	Weakness	Left	L1	70-85	0.75-0.9
2.6-3	Weakness	Left	Celiac trunk	60-75	0.7-0.85

At the celiac trunk, the mean crural thickness falls between 3 mm and 4 mm; men show slightly higher values (approximately 3.2 mm) than women (approximately 2.9 mm). At the L1 vertebral level, the right hemidiaphragm averages approximately 4.3 mm and the left approximately 4 mm, with the maximum pillar thickness reaching 7.4 mm at L1 [17,32].

Diagnostic thresholds derived from CT are summarized in Table 2. These thresholds must be interpreted separately for each side and measurement level because the diaphragmatic crura are inherently asymmetric: the right crus is structurally thicker, longer, and mechanically more robust than the left, reflecting both hepatic support and functional demands [13-16]. This asymmetry produces distinct patterns of pathological thinning. Left-sided paralysis and weakness consistently result in observable reductions in pillar thickness at both L1 and celiac trunk levels, whereas right-sided weakness may not produce sufficient thinning to be identified on CT. Right-sided paralysis is most reliably identified at the L1 level.

The thresholds in Table 2 represent an interpretive synthesis of diagnostic patterns reported in non-myasthenic cohorts by Lallement et al. [19] and are not validated for use in MG. Sensitivity and area under the curve values are expressed as rounded ranges summarizing reported diagnostic performance; original confidence intervals and exact figures are available in the source publication [19]. They are presented for methodological context only and must not be used to guide clinical management of MG patients in the absence of dedicated MG-specific validation.

Reproducibility of measurements

Multiple studies confirm excellent reproducibility of CT-derived diaphragm thickness measurements in non-myasthenic cohorts. In a cohort of 44 patients, the intraclass correlation coefficient (ICC) for measurements at the celiac trunk level was 0.948 (95% CI: 0.918 to 0.967). Intraobserver analysis yielded ICC values ranging from 0.758 to 1 across all measurement points. Axial measurements demonstrated higher interobserver agreement (ICC: 0.886 to 0.926) compared with coronal measurements (ICC: 0.776 to 0.79) [18], favoring the axial plane when single-plane assessment is performed.

Comparable MG-specific reproducibility data are not yet available. Reproducibility of diaphragm ultrasound, however, has been established in MG [23], and equivalent CT validation in MG cohorts is required.

Integration of CT with functional assessment

Optimal respiratory assessment in MG requires integration of multiple modalities. CT establishes the structural basis; ultrasound characterizes dynamic function and can be repeated without radiation exposure; spirometry quantifies integrated output of all respiratory muscles as volumes, flows, and pressures [33,34]. Transdiaphragmatic pressure measurement can provide functional information on diaphragmatic contractility, but its invasiveness prevents widespread clinical applications [9,35].

Diaphragm dysfunction in MG may develop as a relatively isolated phenomenon or may precede detectable spirometric abnormalities. Diaphragmatic weakness can be compensated by accessory respiratory muscle activation, masking functional impairment on standard pulmonary function testing while structural CT abnormalities are already present [9,11,35]. In this setting, CT provides a structural signal that spirometry may not capture.

Monitoring therapeutic response

CT assessment of the diaphragm has been proposed as a candidate tool for monitoring treatment response in MG, but structural monitoring of immunotherapy is not yet validated in MG. Immunosuppressive therapy with glucocorticoids, azathioprine, or mycophenolate mofetil is directed at reducing autoimmune activity and restoring neuromuscular transmission; substantial functional recovery might plausibly be accompanied by measurable structural normalization over time, but this remains a hypothesis [5]. The effects of plasmapheresis and intravenous immunoglobulin on structural diaphragmatic changes have not been adequately studied. However, there are two case reports with diaphragm ultrasound-guided treatment of respiratory failure in patients with myasthenic crisis, with description of all the treatment options [24,27].

Serial CT measurements during treatment could provide observer-independent structural endpoints for clinical trials in MG and may detect subtle changes over time. Radiation dose, however, limits scanning frequency, and ultrasound remains preferable for frequent serial monitoring [23,24,30]. CT may have a complementary role during weaning in severe and complicated disease, as demonstrated for ultrasound by Saishu et al. [27].

Risk stratification for myasthenic crisis

Myasthenic crisis, defined as life-threatening respiratory failure requiring mechanical ventilation, is the gravest complication of MG [8]. Escalating respiratory muscle weakness, traditionally assessed clinically and with spirometry, is the principal herald of crisis. QMGS dynamics as a predictor of post-thymectomy myasthenic crisis, as documented by the current authors, underscores the value of objective biomarkers in this population [12]. CT detection of reduced diaphragm thickness could plausibly contribute an additional structural marker of risk, particularly when spirometry is preserved through compensatory mechanisms, but CT-based crisis prediction is not yet established and should not currently inform clinical decision-making.

Thresholds proposed in other neuromuscular diseases (e.g., total mean diaphragm thickness <3.7 mm as a predictor of intensive care unit admission [3,4]) and the experience in amyotrophic lateral sclerosis, where thickness correlates with disease progression and may guide diaphragm pacing decisions [36,37], require prospective MG-specific validation before any clinical adoption.

Limitations and methodological considerations

Several limitations warrant consideration. First, this review is a narrative synthesis rather than a systematic review with a formal risk of bias assessment. Inclusion criteria were applied consistently but without a formal PRISMA-compliant selection process, and the diagnostic thresholds reported in Table 2 represent an interpretive synthesis that should be confirmed against the original source publications before clinical adoption.

Second, the evidence base for CT diaphragm assessment specifically in MG remains limited. Most published work employs mixed neuromuscular disease cohorts or non-MG populations. Dedicated prospective studies in MG populations with stratification by antibody profile (AChR-positive, MuSK-positive, seronegative), disease severity (MGFA classification), and treatment status are needed.

Finally, the optimal reference standard for defining diaphragm dysfunction in MG remains debated. Ultrasound and fluoroscopy carry their own limitations; transdiaphragmatic pressure measurement represents a more rigorous functional standard but its invasiveness and technical complexity limit application in large-scale studies [9,35].

Proposed research framework for a candidate standardized CT protocol

The acquisition and measurement parameters described below are intended as a proposed research framework to support consistent data collection in future MG-specific studies, rather than as a validated clinical protocol. MG‑specific diagnostic thresholds, prospective MG cohorts, reproducibility studies in MG populations, and outcome‑validation studies will be required before any clinical implementation. Image acquisition should be performed with the patient in the supine position during a breath held at full inspiration, using a 64‑slice or higher multidetector CT scanner. A slice thickness of 1.5 mm or less is recommended to permit high‑quality multiplanar reformation, with axial and coronal reconstructions generated at 1.5 mm intervals. Contrast administration is optional, as both enhanced and non‑enhanced acquisitions are acceptable [17,18].

For measurement, images should be reviewed using mediastinal window settings (center 90 HU; width 350 HU), with magnification adjusted freely to optimize visualization. At the celiac trunk level on axial images, diaphragm thickness should be measured perpendicular to the muscle surface at the point where a horizontal tangent drawn along the anterior spinal canal margin intersects the lateral hemidiaphragm contours, with right and left sides recorded separately [16-18]. At the L1 vertebral level on coronal images, thickness should be measured along the anterior aspect of the L1 mid‑body, perpendicular to the diaphragm surfaces, again recording right and left values independently [16-18].

Structured reporting should include individual thickness measurements with clear anatomical level designation, the total mean diaphragm thickness, and the difference in hemidiaphragm dome heights. CT attenuation values should be documented when available. Measurements should be compared with normative reference ranges and published diagnostic thresholds, followed by a concise conclusion classifying each hemidiaphragm as normal, weak, or paralyzed. When measurements approach threshold values, the report should recommend clinical correlation, and when structural abnormalities are identified, ultrasound confirmation should be advised [16,18,19,23].

## Conclusions

CT evaluation of diaphragm thickness is a promising structural metric that may complement functional respiratory assessment in MG. In non‑myasthenic cohorts, measurements at the L1 vertebral level and celiac trunk show consistent correlations with ultrasound‑derived functional indices, particularly on the left side, and exhibit laterally specific thresholds for weakness and paralysis. Interobserver agreement is good, but MG‑specific reproducibility, normative values, and outcome‑linked thresholds remain unavailable; therefore, clinical application in MG would be premature without dedicated validation.

CT and ultrasound provide synergistic but non‑interchangeable perspectives on diaphragmatic involvement in MG. CT offers an operator‑independent structural assessment suitable for documentation and longitudinal monitoring, whereas ultrasound uniquely captures the dynamic fatigability that characterizes MG. Existing evidence is preliminary, based on small and heterogeneous populations, and does not establish MG‑specific diagnostic criteria or decision rules. The proposed CT framework provides a practical foundation for future prospective studies stratified by serological subtype, MGFA severity, and treatment status, enabling the development of validated MG‑specific thresholds that may ultimately improve prediction of respiratory deterioration and therapeutic response.
